# Nuclear Magnetic Resonance Treatment Accelerates the Regeneration of Dorsal Root Ganglion Neurons *in vitro*

**DOI:** 10.3389/fncel.2022.859545

**Published:** 2022-03-28

**Authors:** Anda Mann, Bibiane Steinecker-Frohnwieser, Aida Naghilou, Flavia Millesi, Paul Supper, Lorenz Semmler, Sonja Wolf, Lena Marinova, Lukas Weigl, Tamara Weiss, Christine Radtke

**Affiliations:** ^1^Department of Plastic, Reconstructive and Aesthetic Surgery, Medical University of Vienna, Vienna, Austria; ^2^Ludwig Boltzmann Institute for Arthritis and Rehabilitation, Saalfelden, Austria; ^3^Austrian Cluster for Tissue Regeneration, Vienna, Austria; ^4^Department of Special Anesthesia and Pain Therapy, Medical University of Vienna, Vienna, Austria

**Keywords:** peripheral nerve regeneration, low nuclear magnetic resonance therapy, Schwann cell, neurite outgrowth, DRG neuron, proliferation

## Abstract

Functional recovery from peripheral nerve injuries depends on a multitude of factors. Schwann cells (SCs) are key players in the regenerative process as they develop repair-specific functions to promote axon regrowth. However, chronically denervated SCs lose their repair phenotype, which is considered as a main reason for regeneration failure. Previous studies reported a modulatory effect of low nuclear magnetic resonance therapy (NMRT) on cell proliferation and gene expression. To provide first insight into a possible effect of NMRT on cells involved in peripheral nerve regeneration, this study investigated whether NMRT is able to influence the cellular behavior of primary SC and dorsal root ganglion (DRG) neuron cultures *in vitro*. The effect of NMRT on rat SCs was evaluated by comparing the morphology, purity, proliferation rate, and expression levels of (repair) SC associated genes between NMRT treated and untreated SC cultures. In addition, the influence of (1) NMRT and (2) medium obtained from NMRT treated SC cultures on rat DRG neuron regeneration was examined by analyzing neurite outgrowth and the neuronal differentiation status. Our results showed that NMRT stimulated the proliferation of SCs without changing their morphology, purity, or expression of (repair) SC associated markers. Furthermore, NMRT promoted DRG neuron regeneration shown by an increased cell survival, enhanced neurite network formation, and progressed neuronal differentiation status. Furthermore, the medium of NMRT treated SC cultures was sufficient to support DRG neuron survival and neurite outgrowth. These findings demonstrate a beneficial impact of NMRT on DRG neuron survival and neurite formation, which is primarily mediated via SC stimulation. Our data suggest that NMRT could be suitable as a non-invasive auxiliary treatment option for peripheral nerve injuries and encourage future studies that investigate the effect of NMRT in a physiological context.

## Introduction

Peripheral nerve injuries affect up to 2.8% of trauma patients and represent a therapeutic challenge with still unsatisfactory functional outcome (Ciaramitaro et al., 2010; Yegiyants et al., 2010). Although peripheral nerves possess an inherent capacity to regenerate after minor injuries such as segmental demyelination or axonotmesis, recovery is poor when axons have to regrow over long distances or face the challenge to overcome nerve gaps (SEDDON, 1943).

On a cellular level, nerve regeneration is largely dependent on Schwann cells (SCs), the principal glia of the peripheral nervous system (Arthur-Farraj et al., 2012). After nerve damage, SCs convert into dedicated repair cells and perform a variety of tasks specialized to the needs of injured nerves (Griffin and Thompson, 2008; Arthur-Farraj et al., 2012). The repair SC phenotype is characterized by the re-expression of genes involved in SC development and the acquisition of distinctive repair−supportive functions such as myelin and debris clearance and the production of chemokines and cytokines (Rutkowski et al., 1999; Gomez-Sanchez et al., 2015; Jang et al., 2016; Jessen and Mirsky, 2016; Weiss et al., 2016; Peng et al., 2020). In addition, repair SCs proliferate, regain a migratory potential, and align within their basal lamina tubes to form regeneration tracks (bands of Büngner) that provide orientation and spatial cues for the sprouting axons (Gomez-Sanchez et al., 2017; Jessen and Arthur-Farraj, 2019). Repair SCs also express a variety of neuritogenic and neurotrophic factors that ensure the survival and promote the re-growth of injured axons (Gordon, 2009). In the final stage of regeneration, SCs redifferentiate into their adult phenotype to restore nerve function.

However, nerve injuries far away from the target organ challenge the axons to regenerate over long distances. As a consequence, repair SCs residing in the very distal nerve segment experience prolonged lack of axon contact. These chronically denervated SCs have been shown to lose their repair phenotype and gradually reduce proliferation followed by cell death (Fu and Gordon, 1995; Höke, 2006; Painter, 2017; Jessen and Mirsky, 2019). This phenomenon is referred to as denervation-induced SC senescence and leads to a permanent functional deficit (Höke, 2006; Sulaiman and Gordon, 2009; Jessen and Mirsky, 2019). Hence, current research aims to prolong the time window for nerve regeneration by finding novel ways to sustain the repair phenotype of SCs and stimulate axon outgrowth over an extended time period.

Nuclear magnetic resonance (NMR) is a physical effect that results from the magnetic properties of certain atomic nuclei. The principle involves three steps (1) the alignment of atomic nuclear spins in a constant magnetic field, (2) the perturbation of that alignment by a weak oscillating magnetic field (radio frequency pulse), and (3) the realignment with the magnetic field, which produces an NMR signal characteristic for the respective atomic nucleus (Marion, 2013). NMR is routinely exploited in advanced medical imaging techniques, such as in magnetic resonance imaging (field intensity 0.2–5.0 T, radiofrequency 10–200 MHz). In the last decades, low intensity nuclear magnetic resonance therapy (NMRT) gained attention due to its positive effects on patients suffering from musculoskeletal disorders and severe back pain (field intensity 0.4–3 mT, radiofrequency 17–130 kHz) (Kullich et al., 2006, 2013). *In vitro* studies analyzed the cellular response of different cell types to NMRT and demonstrated diverse consequences. NMRT enhanced the proliferation of chondrocyte and osteoblast cell cultures but did not affect apoptosis or viability (Temiz-Artmann et al., 2005). In chondrocytes, NMRT counteracted IL-1β mediated signaling events and modulated the expression of growth factors and miRNAs (Steinecker-Frohnwieser et al., 2014, 2021). Skin fibroblasts exposed to NMRT produced less cross-linked collagen and showed changes in the expression of proteins involved in cell adhesion and movement (Digel et al., 2007). In zebrafish fibroblast cells NMRT modulated the expression and oscillation of specific core clock genes and Hif isoforms independently from the known light-induced effects (Oliva et al., 2019).

This study aimed to investigate whether NMRT also affects the cellular behavior of peripheral nerve cells to assess a therapeutic potential for nerve injuries. To this end, we used primary rat SC and DRG neuron cultures, which are established models to study nerve regeneration, and performed a detailed characterization of their cellular features in response to NMRT *in vitro*. The results provide first data on the influence of NMRT on cells involved in neural regeneration.

## Materials and Methods

### Animals

Sciatic nerves, and lumbar dorsal root ganglia (DRGs) were harvested from female and male 20–25 weeks old Sprague-Dawley rats for SC and sensory neuron isolation. The sacrifice of the animals was conducted by deep isoflurane narcosis before guillotine decapitation. The breeding, housing, transport, and sacrifice of animals was conducted in compliance with the Austria’s Animal Testing Law (TVG 2012, §2, 1.c) and Article 3 of the Directive 2010/63/EU of the European Parliament and of the Council on the Protection of Animals Used for Scientific Purposes (European Parliament, 2010).

### Isolation and Culture of Rat Schwann Cells for Nuclear Magnetic Resonance Therapy Treatment

**FIGURE 1:**
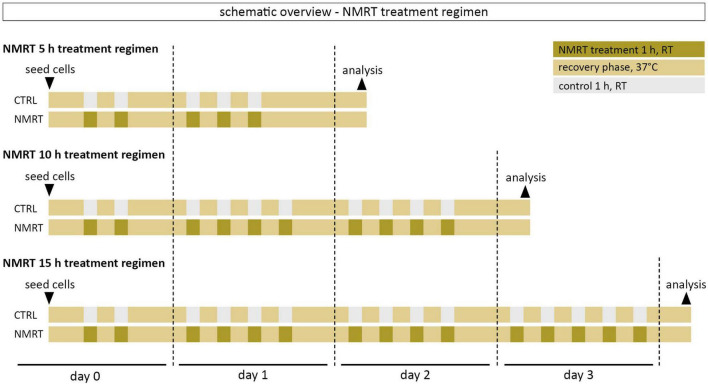
Schematic overview of the NMRT treatment regimen. The first NMRT treatment cycle was conducted 5 h after seeding of cells.

The culture of rat SCs was performed as described before (Haertinger et al., 2020; Weiss et al., 2020). Briefly, sciatic nerves were excised and washed in 1x Dulbecco’s Phosphate Buffered Saline (1x PBS, GIBCO, Waltham, MA, United States) + 1% antibiotic-antimycotic (Thermo Fisher Scientific, Waltham, MA, United States). The fascicles were pulled out of the epineurium and were digested overnight in MEM∝ (Minimum Essential Medium alpha, GIBCO, Waltham, MA, United States) supplemented with 10% fetal calf serum (FCS, LINARIS, Wertheim Bettingen, Germany), 1% Penicillin-Streptomycin (P/S, GIBCO, Waltham, MA, United States), 1% Sodium Pyruvate Solution (NaP, GIBCO, Waltham, MA, United States), 2.5% 4-(2-hydroxyethyl)-1-piperazineethanesulfonic acid buffer solution (HEPES, Sigma-Aldrich, Burlington, MA, United States), 0.125% (w/v) collagenase type IV (GIBCO, Waltham, MA, Unites States), 1.25 U/ml Dispase II (Sigma-Aldrich, Burlington, MA, United States) and 3 mM calcium chloride (Merck, Kenilworth, NJ, United States) at 37°C and 5% CO_2_. The next day, cells were washed and seeded on 0.01% poly-L-lysine hydrobromide (PLL, Sigma-Aldrich, Burlington, MA, United States) and 5 μg/ml laminin (Sigma-Aldrich, Burlington, MA, United States) coated dishes in SC culture medium (SCM) consisting of MEM∝ supplemented with 1% P/S, 1% NaP, 2.5% HEPES, 0.5% N-2 Supplement (GIBCO, Waltham, MA, Unites States), 2 μM forskolin (Sigma-Aldrich, Burlington, MA, United States), 10 ng/ml recombinant Heregulin ß-1 (PeproTech, Rocky Hill, NJ, United States), 10 ng/ml recombinant FGF-basic (fibroblast growth factor basic, PeproTech, Rocky Hill, NJ, United States), 5 ng/ml PDGF-AA (platelet-derived growth factor, PeproTech, Rocky Hill, NJ, United States) and 5% FCS. Separation of SCs from fibroblasts was achieved during passaging using a two-step enrichment procedure (Weiss et al., 2018), which usually resulted in a SC culture purity of over 95% (Weiss et al., 2020). SCs were passaged upon reaching 80–90% confluence and cultures from passage 2 (p2), but not higher than p6 were used for these experiments. Half of the medium was changed three times a week. SCs (1 × 10^4^/cm^2^) were seeded on PLL/laminin coated wells of 8-well μ-slide (ibidi, Gräfelfing, Germany) or T25 flask (GIBCO, Waltham, MA, United States) in SCM and allowed to attach 5 h before NMRT or control treatment (see Figure 1). SCs grown in 8-well μ-slides were immunostained for purity and proliferation analysis, SCs grown in T25 flasks were used for supernatant harvest and gene expression analysis.

### Isolation and Culture of Dorsal Root Ganglion Neurons for Nuclear Magnetic Resonance Therapy Treatment

DRG neurons were cultured as described before (Millesi et al., 2021). Briefly, 3–4 lumbar DRG pairs (L3–L6) were harvested from the spinal roots and washed in 1x PBS + 1% antibiotic-antimycotic. DRGs were cut into smaller pieces and digested overnight using the same digestion solution as described for the nervous tissue (see above). The next day, the pooled cell suspension was washed, resuspended in 1 ml Neurobasal™-A medium (GIBCO, Waltham, MA, United States) and centrifuged through a 20% Percoll solution (Merck, Kenilworth, NJ, United States). The pellet was resuspended in 500 μl DRG medium (DRGM) consisting of Neurobasal™-A medium supplemented with 10 ng/ml recombinant NGF (Invitrogen, Waltham, MA, United States), 1x B27 supplement (Invitrogen, Waltham, MA, United States), 2 mM L-glutamine (Invitrogen, Waltham, MA, United States) and 1% P/S. 20 μl of the DRG cell suspension was plated per 8 well of a 0.01% PDL and 5 μg/ml laminin coated 8-well μ-slide. The DRG cell suspension was allowed to adhere for a period of 5 h before 10 h of NMRT or control treatment (see Figure 1). DRG cultures were then immunostained for neurite feature analysis.

Phase contrast images of SC and DRG cultures were regularly taken with a benchtop microscope (NIKON Eclipse Ts2R, Tokyo, Japan).

### Nuclear Magnetic Resonance Therapy Treatment Regimen

Cells were treated with a therapeutic low intensity NMR device adapted for cell cultures (MBST, MedTec Medizintechnik GmbH, Wetzlar, Germany, mean dynamic magnetic field strength of the sweep). The device parameters were chosen based on a previous *in vitro* study demonstrating favorable effects on cells to a field of 0.4 mT and a radiofrequency of 16 kHz (Oliva et al., 2019). One treatment cycle consisted of 1 h NMRT at ambient conditions followed by a 1.5 h recovery phase in the incubator at 37°C and 5% CO_2_. The 5 h treatment involved five treatment cycles: 2 cycles on day 0 and 3 cycles on day 1. The 10 h treatment involved 10 treatment cycles: 2 cycles on day 0, 4 cycles on day 1, and 4 cycles on day 2. The 15 h treatment involved 15 treatment cycles: 2 cycles on day 0, 4 cycles on day 1, 4 cycles on day 2, and 5 cycles on day 3. The control cultures were also kept at ambient conditions for the respective NMRT treatment time. After the last treatment cycle of the respective condition, cells were incubated at 37°C and 5% CO_2_ overnight before further analysis. A schematic overview of the NMRT treatment regimen can be found in Figure 1.

### Proliferation Assay

The proliferative effect of NMRT on SCs was assessed by visualizing DNA synthesizing cells using the Click–iT Plus EdU Alexa Fluor 555 Imaging Kit (Invitrogen, Waltham, MA, United States). SCs were cultured in SCM and treated according to the 5, 10, and 15 h NMRT treatment regimen. The day after the last treatment cycle, 10 μM EdU was added to the SC cultures and incubated for 2 h. EdU detection was performed according to the manufacturer’s protocol.

### Immunofluorescence Staining and Analysis

The staining procedure was carried out at room temperature unless otherwise noted. A washing step involved a sequential incubation with 1x PBS for 5 min each. All antibodies and details are listed in Supplementary Table 1. Before the immunofluorescence staining, NMRT treated and untreated (control) SCs and DRG neurons were washed with 1x PBS and fixed with 4.5% formaldehyde solution (SAV Liquid Production GmbH, Flintsbach am Inn, Germany) for 15 min. Blocking and permeabilization was performed in one step with 1x PBS containing 1% bovine serum albumin (BSA, Sigma-Aldrich, Burlington, MA, United States), 0.3% Triton X-100 (Sigma-Aldrich, Burlington, MA, United States), and 5% goat serum (DAKO, Glostrup, Denmark) for 15 min. Subsequently, the cells were incubated with primary antibodies diluted in staining solution (1x PBS containing 1% BSA, 0.1% Triton X-100, and 1% goat serum) overnight at 4°C. The next day, cells were washed and incubated with respective secondary antibodies diluted in the staining solution for 1 h. For nuclear staining, 50 μg/ml 4,6-Diamidino-2-Phenylindole solution (DAPI, Thermo Fisher Scientific, Waltham, MA, United States) was added for 10 min. After washing, the cells were embedded in Fluoromount-G mounting medium (Invitrogen, Waltham, MA, United States). Immunofluorescence images were taken using an Eclipse Ti (Nikon, Tokyo, Japan) microscope. Images from one donor in different conditions were acquired at the same day with the same settings to enable comparative image analysis. Images are depicted as maximum intensity projections of total z-stacks, contrast and brightness have been adapted in a homogenous manner, and pseudo coloring was applied to make multicolor figures comprehensible for color-blind readers.

### Quantification of EdU Positive Cells

For the quantification of proliferating SCs, the CellCounter plugin of ImageJ 1.47^1^ was used. At least 300 DAPI^+^ nuclei were counted per experimental condition by excluding burst nuclei and nuclei cut by the image boarder. Subsequently, SOX10^+^/DAPI^+^ nuclei (SCs) and SOX10^+^/DAPI^+^/EdU^+^ nuclei (proliferating SCs) were counted.

### Quantification of Dorsal Root Ganglion Numbers and Neurite Features

The total number of DRG neurons was evaluated by manual counting of neuronal cell bodies that grew within one well of an 8-well chamber slide without and with NMRT treatment (*n* = 4). For the quantification of neurite features (primary neurites, branching points, neurite length), DRG cultures were immunostained for tubulin β-3 and 15–20 DRG neurons were analyzed in control and NMRT treated DRG cultures, respectively, of 4 individual donors (*n* = 4). The number of primary neurites per DRG neuron and mean number of branching points per DRG neurons were determined by manual counting. The mean length of all neurites per DRG neuron visible per field of view was evaluated using the “free line tool” of ImageJ. The mean neurofilament fluorescence staining intensity of NMRT treated and control DRG cultures (*n* = 4) was calculated using the “measure” function of ImageJ.

### Reverse Transcription Polymerase Chain Reaction

RNA isolation of NMRT treated and untreated SCs was performed with the RNeasy kit (Qiagen, Hilden, Germany) and DNase-I (Qiagen, Hilden, Germany) according to the manufacturer’s manual. Subsequently, RNA concentration was measured with a Nanophotometer (Implen, Munich, Germany) and 50 ng RNA was reverse transcribed (iScript™ cDNA Synthesis Kit, Biorad, Hercules, CA, United States). The Reverse Transcription Polymerase Chain Reaction (RT-PCR) was performed using SYBR Green Supermix (BioRad, Hercules, CA, United States) and the 7500 Fast Real-Time PCR System (Applied Biosystems, Waltham, MA, United States). The respective primer sequences are listed in Supplementary Table 2. Reactions were performed in duplicates. The quantities of target gene expression were calculated relative to the geometric mean of the internal control genes, GAPDH and β-actin, and presented as gene expression fold change between NMRT treated and control samples [2^–(ΔΔCT)^].

### Medium Experiments

**FIGURE 2:**
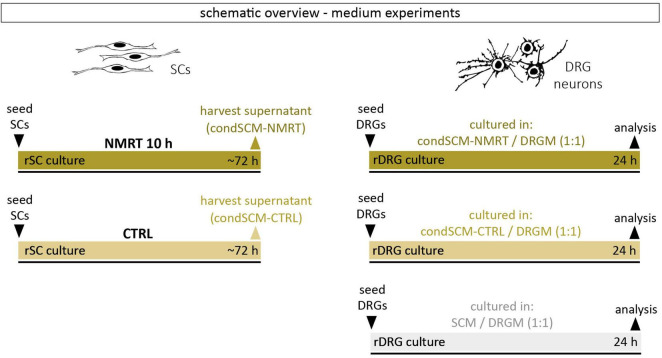
Schematic overview of medium experiments.

Conditioned medium of SC cultures after a 10 h NMRT treatment (condSCM-NMRT) and of untreated control SC cultures (condSCM-CTRL) was harvested and mixed 1:1 with DRG culture medium (DRGM) without NGF, respectively. SC medium (SCM) mixed 1:1 with DRGM without NGF served as additional control. Freshly dissociated DRGs were seeded in the condSCEM-NMRT/DRGM, the condSCEM-CTRL/DRGM and the SCM/DRGM media, respectively, and cultured for 24 h before the analysis of neurite features. A schematic overview of the medium experiment set-up can be found in Figure 2.

### Statistical Analysis

The normality of data distribution was assessed by quantile-quantile plots and data were logarithmized. The significance of differences of the measured parameters between experimental conditions were evaluated with Wolfram Mathematica using a two-way ANOVA approach. Two-way ANOVA allows the estimation of the impact of two different categorical independent variables on one continuous dependent variable (Norton and Strube, 1986), here, the impact of the experimental condition and the cell donors on the measured parameter values. An additional statistical analysis was performed with GraphPad Prism 8 using a grouped analysis two-way ANOVA. The donor dependent visualization of measured parameter values for statistical analysis is shown in Supplementary Figure 1. The results are depicted as single values for each donor ± standard deviation (SD); *p < 0.05, ^**^p < 0.01, ^***^p < 0.001.

## Results

### Nuclear Magnetic Resonance Therapy Stimulated Schwann Cell Proliferation but Had No Effect on Schwann Cell Morphology

**FIGURE 3:**
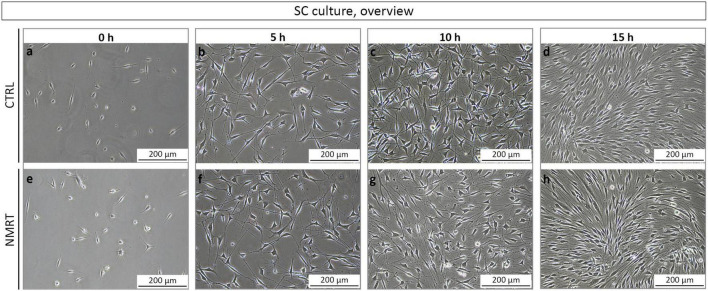
Morphological analysis of SC cultures in response to NMRT. Representative phase contrast images illustrating a similar morphology of **(a–d)** control SC cultures (upper images) and **(e–h)** NMRT treated SC cultures (lower images) before treatment (0 h = 5 h after seeding) and after the 5, 10, and 15 h treatment cycles.

In order to analyze and compare the morphology between control and NMRT treated SC cultures, phase contrast images from all conditions were taken before treatment and after the 5, 10, and 15 h treatment regimen. In all conditions, SCs possessed a spindle-shaped morphology with di- and tri-polar extensions and formed the typical swirled parallel alignment with increased culture density (Figures 3a–h), no changes were noted in the NMRT treated group in comparison to the control group. Thus, NMRT did not affect the morphology of SCs or their capability to align to each other.

### Nuclear Magnetic Resonance Therapy Did Not Affect Schwann Cell Purity or the Expression of Repair Schwann Cell Associated Genes

**FIGURE 4:**
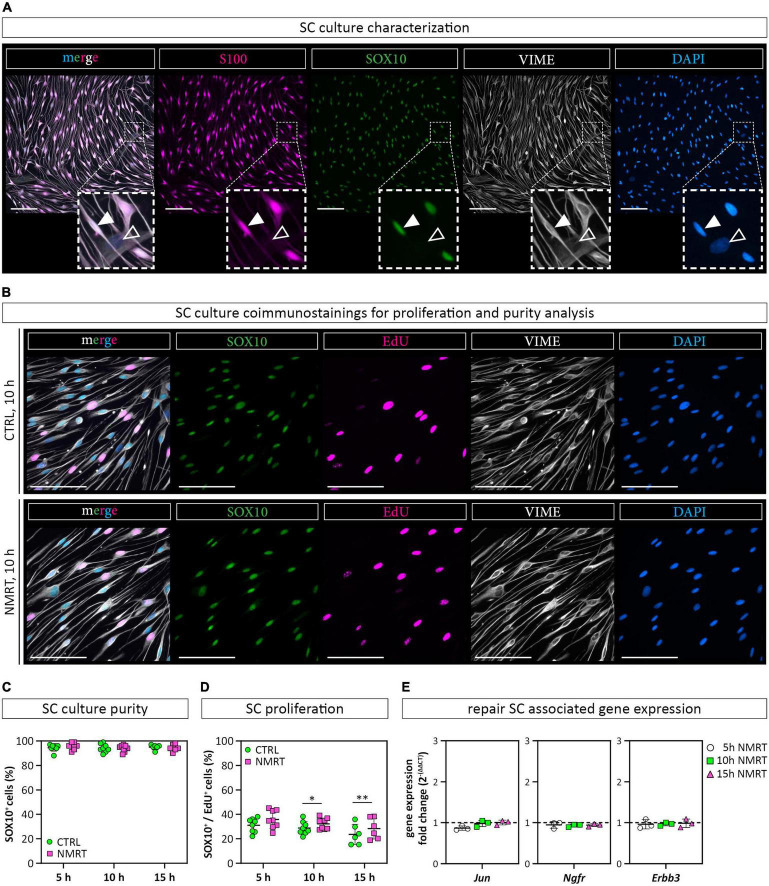
Comparison of SC behavior in response to NMRT. **(A)** Representative fluorescence images of a primary SC culture stained for SC markers SOX10 (green) and S100 (magenta) as well as intermediate filament vimentin (VIME, white) and DAPI (blue); filled arrowheads indicate a SOX10^+^/S100^+^/VIME^+^/DAPI^+^ SC, lined arrowheads indicate a SOX10^–^/S100^–^/VIME^+^/DAPI^+^ fibroblast. Scale bars represent 100 μm. **(B)** Representative fluorescence images of control SC cultures (upper panel) and NMRT treated SC cultures (lower panel) after the 10 h NMRT treatment cycle stained for SC marker SOX10 (green), proliferation marker EdU (magenta), VIME (white) and DAPI (blue). Scale bars represent 50 μm. Representative fluorescence images after the 5 and 15 h treatment cycles are shown in Supplementary Figure 2. **(C)** SC culture purity was calculated in percent of SOX10^+^/DAPI^+^ cells (SCs) from DAPI^+^ cells (all cells) and showed no difference between the NMRT and CTRL condition; data are depicted as single values for each donor ± SD (*n* = 6). **(D)** Proliferating SCs were calculated in percent of SOX10^+^/EdU^+^/DAPI^+^ cells (proliferating SCs) from SOX10^+^/DAPI^+^ cells (SCs) showing an increase of EdU^+^ SCs in NMRT treated cultures after the 10 and 15 h treatment cycle compared to respective controls; data are depicted as single values for each donor ± SD, 5 h (*n* = 8), 10 h (*n* = 8), 15 h (*n* = 6), two-way ANOVA, *p < 0.05, ^**^p < 0.01. **(E)** Diagrams show the RT-PCR results of repair SC associated genes *Jun*, *Ngfr*, and *Erbb3* as the gene expression fold change [2^–(ΔΔ*CT*)^] between NMRT treated and control samples; data are depicted as single values for each donor ± SD (*n* = 3), two-way ANOVA.

Next, we used a well-established multicolor immunofluorescence staining analysis (Weiss et al., 2020, 2021) to qualitatively and quantitatively assess whether the NMRT treatment influences the purity or proliferation of SC cultures over time. The SC cultures were characterized by the expression of the S100 protein and the glial cell associated transcription factor SOX10 demonstrating a highly enriched SC population (Figure 4A). The few fibroblasts present in SC cultures are negative for S100 and SOX10, while both SCs and fibroblasts express the intermediate filament vimentin (VIME) (Figure 4A). As the nuclear staining of SOX10 is more convenient for image analysis, the following proliferation assay used SOX10 and not S100 for the determination of SC identity. DNA synthesizing cells were visualized by EdU detection. The VIME staining was used to show all cells. Coimmunostainings and EdU detection of SC cultures were performed after the 5 h (Supplementary Figure 2A), 10 h (Figure 4B) and 15 h (Supplementary Figure 2B) NMRT treatments alongside with respective controls. The immunofluorescence images illustrated SOX10 positive SCs, EdU positive nuclear signals in DNA synthesizing cells, and VIME positive cytoskeletal constituents. The number of SCs (SOX10 positive cells) and proliferating SCs (SOX10 and EdU positive cells) were quantified for each condition. The results showed a purity of about 95% in both control and NMRT treated SC cultures after the 5 h (CTRL: 94.50 ± 2.83, NMRT: 5 h 95.88 ± 2.85), 10 h (CTRL: 94.13 ± 3.40, NMRT: 93.88 ± 2.75) and the 15 h (CTRL: 95.00 ± 2.10, NMRT: 94.33 ± 2.88) treatments (Figure 4C). The amount of proliferating SCs was similar in the 5 h control and NMRT groups (CTRL: 31.04 ± 5.74, NMRT: 35.80 ± 7.34). Notably, proliferation was slightly but significantly increased in the 10 h NMRT group (CTRL: 28.83 ± 5.36, NMRT: 32.21 ± 4.58) and 15 h NMRT group (CTRL: 23.58 ± 8.17, NMRT: 30.04 ± 7.55) (Figure 4D). We further tested whether NMRT affects the mRNA expression levels of repair SC associated tumor necrosis factor receptor superfamily member 16 (*Ngfr*), receptor tyrosine-protein kinase erbB-3 (*Erbb3*) and transcription factor AP-1 (*Jun).* No difference between control and NMRT treated SC cultures were found for the expression of *Ngfr* (CTRL: 1, NMRT 5 h: 0.95 ± 0.08; NMRT 10 h: 0.93 ± 0.03; NMRT 15 h: 0.94 ± 0.04), *Erbb3* (CTRL: 1; NMRT 5 h: 0.97 ± 0.11; NMRT 10 h: 0.97 ± 0.04; NMRT 15 h: 0.99 ± 0.11) and *Jun* (CTRL: 1, NMRT 5 h: 0.87 ± 0.05; NMRT 10 h: 0.98 ± 0.08; NMRT 15 h: 1.00 ± 0.06) (Figure 4E). These findings show that the purity of SCs and the expression of repair SC associated genes was similar in NMRT treated and untreated SC cultures. However, NMRT slightly enhanced the proliferation rate of cultured SCs.

### Nuclear Magnetic Resonance Therapy Enhanced the Attachment and Neurite Outgrowth of Dorsal Root Ganglion Neurons

**FIGURE 5:**
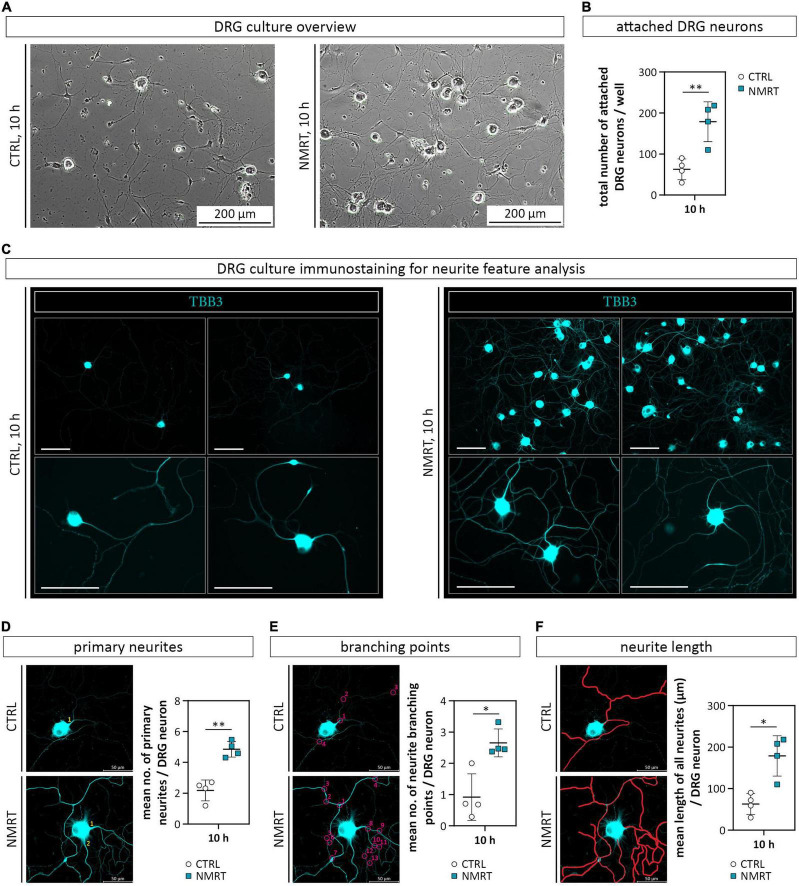
Comparison of DRG cultures and neurite features in response to NMRT. **(A)** Representative phase contrast images illustrating the morphology of control DRG culture (left image) as well as NMRT treated DRG cultures (right image) after the 10 h treatment cycle. **(B)** Counting of the total number of attached DRG neurons per well showed a significant increase of DRG neurons in NMRT treated cultures; data are depicted as mean values for each donor ± SD (*n* = 4), **p < 0.01. **(C)** Representative immunofluorescence images of control DRG cultures (left panel) and NMRT treated DRG cultures (right panel) after the 10 h treatment cycle stained for early neuronal marker tubulin β-3 (TBB3, cyan). An enhanced neurite outgrowth of DRG neurons was observed upon NMRT treatment. Scale bars represent 100 μm. Representative images of TBB3 stained neurons in CTRL and upon NMRT alongside with quantification of **(D)** the mean number of primary neurites per DRG neuron (primary neurites indicated by numbers), **(E)** the mean number of neurite branching points (indicated by numbered circles) per DRG neuron, and **(F)** the mean length of all neurites (indicated by manually drawn neurites in red) per DRG neuron between control and NMRT treated DRG cultures; data are depicted as single values for each donor ± SD (*n* = 4), two-way ANOVA, ^**^p < 0.01, *p < 0.05.

We further investigated if NMRT exerts an effect on primary DRG neurons. To determine early differences in neuronal behavior, we chose the 10 h NMRT treatment regimen for further investigations. Five hours after the seeding of digested DRGs, the cultures were either exposed to NMRT or left untreated. The subsequent phase contrast images suggested that the treated cultures contained more neurons with a denser network of neurites (Figure 5A). Counting of all attached neuronal cell bodies per well confirmed a significantly increased number of DRG neurons after NMRT (CTRL: 62.75 ± 20.59, NMRT: 178.75 ± 48.72) (Figure 5B). For qualitative assessment of the neurite outgrowth, we stained the DRG cultures for the axonal protein tubulin β-3 (TBB3), typically expressed in developing axons. The comparison between control and NMRT treated DRG cultures visualized an advanced neurite network in the latter (Figure 5C). Quantification of neurite features further demonstrated that NMRT treated neurons showed significantly increased numbers of primary neurites (CTRL: 2.18 ± 0.68, NMRT: 4.86 ± 0.51) (Figure 5D) and neurite branching points (CTRL: 0.92 ± 0.75, NMRT: 2.66 ± 0.45) (Figure 5E) alongside with longer neurites (CTRL: 247.49 ± 20.59 μm, NMRT: 505.01 ± 71.26 μm) (Figure 5F). These results indicate that the NMRT treatment had beneficial effects on the survival and neurite network formation of DRG neurons.

### Nuclear Magnetic Resonance Therapy Supported Neuronal Maturation of Dorsal Root Ganglion Neurons

**FIGURE 6:**
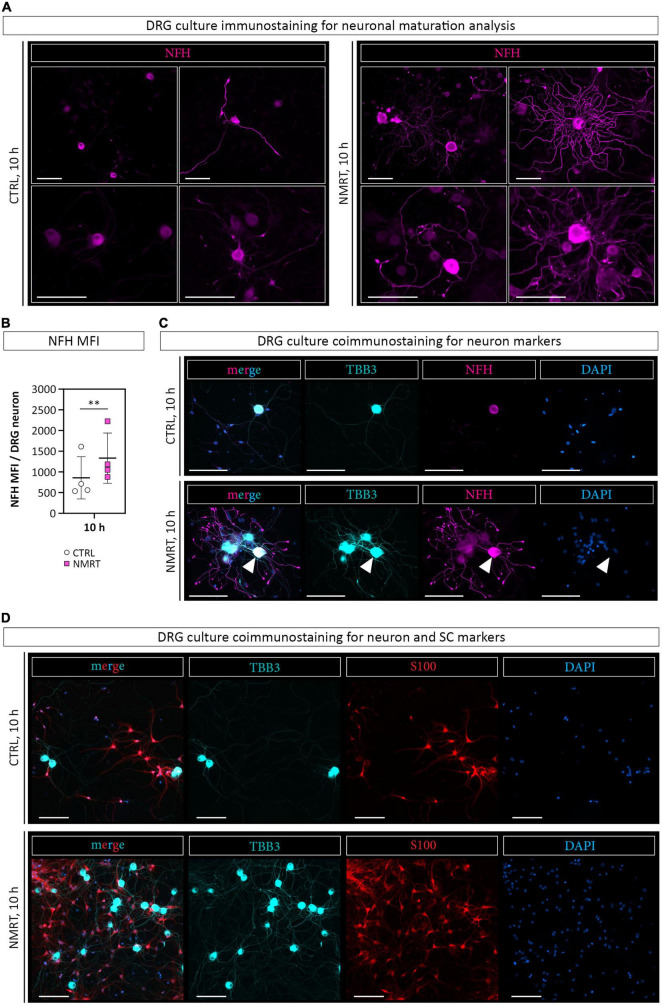
Comparison of neuronal differentiation in response to NMRT. **(A)** Representative immunofluorescence images of control DRG cultures (left panel) and NMRT treated DRG cultures (right panel) after the 10 h treatment cycle stained for axonal maturation marker neurofilament heavy polypeptide (NFH, magenta). Scale bars represent 100 μm. **(B)** Quantification of the NFH mean fluorescence intensity (MFI) per DRG neuron; data are depicted as single values for each donor of ± SD (*n* = 4), two-way ANOVA, ^**^p < 0.01. **(C)** Representative immunofluorescence images of control DRG cultures (upper panel) and NMRT treated DRG cultures (lower panel) after the 10 h treatment regimen stained for TBB3 (cyan), NFH (magenta), and DAPI (blue). Scale bars represent 100 μm. Note that all neuronal cell bodies and neurites are positive for TBB3, while only one neuron expresses NFH in the cell body and neurites (arrowhead). **(D)** Representative immunofluorescence images of control DRG cultures (upper panel) and NMRT treated DRG cultures (lower panel) after the 10 h treatment cycle stained for TBB3 (cyan), S100 (red), and DAPI (blue). An increased number of neurons and SCs is present in the NMRT treated cultures. Scale bars represent 100 μm.

As an increased neurite outgrowth could suggest progressing neuronal maturation, we stained the DRG cultures for neurofilament heavy polypeptide (NFH), a marker for maturing axons. The immunofluorescence images illustrated that NMRT treated cultures contained neurons expressing NFH in the cell body and the neurite network (Figure 6A). Quantification confirmed a significantly higher NFH mean fluorescence intensity (MFI) per DRG neuron in the NMRT treated cultures (CTRL: 855.19 ± 509.99, NMRT: 1333.24 ± 605.99) (Figure 6B). Coimmunostaining with TBB3 and NFH further revealed that not all TBB3 positive neurons express NFH (Figure 6C). The results demonstrated that exposure to NMRT increased the attachment and neurite outgrowth of all neurons and enhanced neuronal maturation of individual neurons.

### Conditioned Medium Derived From Nuclear Magnetic Resonance Therapy Treated Schwann Cell Cultures Was Sufficient to Increase the Survival and Neurite Outgrowth of Dorsal Root Ganglion Neurons

In addition to the elevated number of DRG neurons in NMRT treated cultures, an increase in non-neuronal cells could be observed. Based on the morphology of these cells, and not caring out a SC depletion step in the DRG culture protocol, we assumed that most of the non-neuronal cells represent SCs. Coimmunostaining of NMRT treated and untreated DRG cultures for the SC marker S100 and the neuronal marker TBB3 confirmed that the majority of non-neuronal cells in the DRG cultures indeed are SCs (Figure 6D). Therefore, we next investigated whether the beneficial effect of NMRT on DRG neurons could have been indirectly mediated by the increased number of SCs present in these cultures. SCs are known to produce neuritogenic and neurotrophic factors supporting neuron survival and neurite outgrowth. Hence, we analyzed if the conditioned medium derived from NMRT treated (condSCM-NMRT) and control (condSCM-CTRL) SC cultures would mimic the effect of direct NMRT treatment. As the SC medium contains growth factors that might affect DRG neuron behavior, SC medium (SCM-CTRL) was used as an additional control. To this end, freshly dissociated DRGs were seeded in normal DRG medium (DRGM) without NGF mixed 1:1 with the different SC media (condSCM-NMRT, condSCM-CTRL, and SCM-CTRL) and cultured for 24 h. A schematic overview of the medium experiment set-up can be found in Figure 2.

**FIGURE 7:**
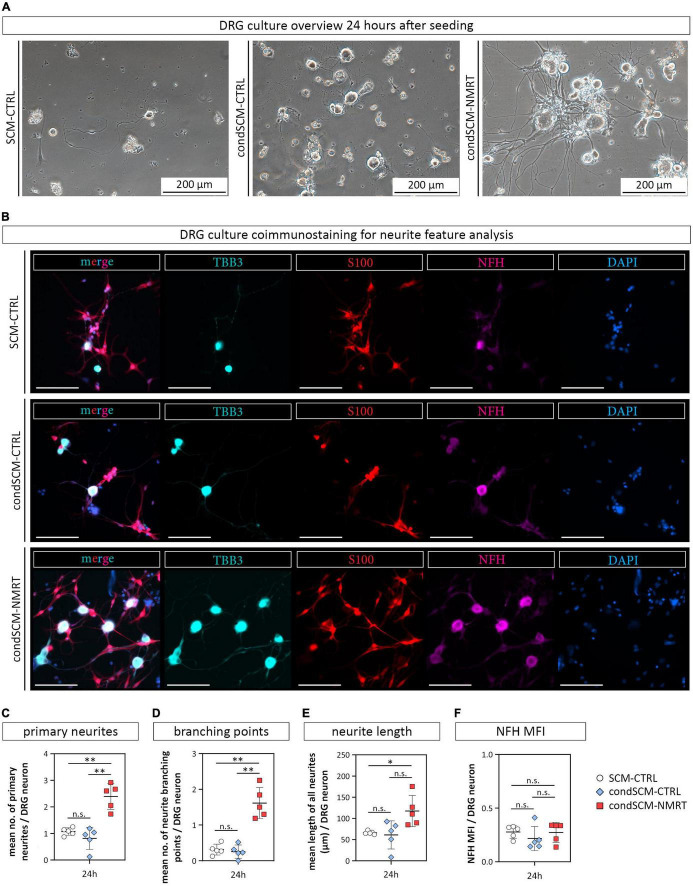
Comparison of DRG cultures and their neurite features in response to conditioned medium derived from NMRT treated SC cultures. **(A)** Representative phase contrast images show DRG cultures 24 h after seeding in either SCM-CTRL (left image), condSCM-CTRL (middle image), and condSCM-NMRT (right image). **(B)** Representative immunofluorescence images of DRG cultures 24 h after seeding in SCM-CTRL (upper panel), condSCM-CTRL (middle panel), and condSCM-NMRT (lower panel) stained for TBB3 (cyan), S100 (red), NFH (magenta), and DAPI (blue). Scale bars represent 100 μm. Quantification of **(C)** the mean number of primary neurites per DRG neuron, **(D)** the mean number of neurite branching points per DRG neuron, **(E)** the mean length of neurites per image (field of view), and **(F)** NFH mean fluorescence intensity (MFI) per DRG neuron **(F)**; data are depicted as single values for each donor ± SD (*n* = 5), two-way ANOVA, ^**^p < 0.01, *p < 0.05, n.s. not significant.

Phase contrast images showed that DRG neurons grew best when cultured with condSCM-NMRT as illustrated by the increased attachment and survival of cells, especially large diameter neurons (Figure 7A). Coimmunostainings for S100, TBB3 and NFH visualized DRG neurons with extended neurites and associated SCs in the condSCM-NMRT, while neurite outgrowth was poor in condSCM-CTRL and SCM-CTRL (Figure 7B). In line with these observations, quantification of neurite features identified that the numbers of primary neurites (SCM-CTRL: 1.07 ± 0.14, condSCM-CTRL: 0.82 ± 0.41, condSCM-NMRT: 2.39 ± 0.49) (Figure 7C), neurite branching points (SCM-CTRL: 0.31 ± 0.15, condSCM-CTRL: 0.256 ± 0.19, condSCM-NMRT: 1.616 ± 0.43) (Figure 7D), and neurite length (SCM-CTRL: 66.16 ± 3.97 μm, condSCM-CTRL: 61.08 ± 33.35 μm, condSCM-NMRT: 117.43 ± 36.48 μm) (Figure 7E) was significantly increased in the cultures exposed to condSCM-NMRT. However, the expression of NFH was similar in all conditions (SCM-CTRL: 0.28 ± 0.06, condSCM-CTRL: 0.22 ± 0.11, condSCM-NMRT: 0.27 ± 0.09) (Figure 7F). These results indicate that the enhanced survival and neurite outgrowth observed in NMRT treated DRG cultures is to a significant extent mediated by secreted molecules produced by SCs in response to NMRT.

## Discussion

The remarkable plasticity of SCs allows them to rejuvenate and adopt an alternative phenotype after nerve injury that is essential for the regeneration of injured peripheral nerves. However, the repair SC phenotype fades when the SCs are chronically deneravted. As NMRT was previously demonstrated to modulate cellular behaviors such as proliferation and gene expression (Temiz-Artmann et al., 2005; Digel et al., 2007; Steinecker-Frohnwieser et al., 2014, 2021; Oliva et al., 2019), this *in vitro* study investigated the potential effect of NMRT on the main cell types involved in peripheral nerve regeneration, SCs and DRG neurons. The results showed a slight NMRT induced increase in SC proliferation, while the morphology and purity of SC cultures remained unchanged, suggesting that NMRT stimulates SCs without inducing an abnormal cell behavior. Interestingly, the expression levels of repair SC associated genes like *Jun*, *Erbb3*, and *Ngfr* remained similar in treated and untreated SCs. As we used early passage SC cultures that represent SCs in their repair state (Weiss et al., 2016), the influence of NMRT on these repair associated genes might be negligible. However, future studies on senescent SCs, which have been shown to downregulate repair associated genes (Jessen and Mirsky, 2019), could reveal whether NMRT is able to rescue SCs from denervation induced senescence.

Furthermore, we observed a strong impact of NMRT on the cellular behavior of rat DRG cultures. The exposure of freshly seeded DRG cultures to a 10 h NMRT treatment significantly increased the number of attached neurons and induced neurite network formation quantified by distinct neurite features such as the number of primary neurites, the number of neurite branching points, and the total neurite length per DRG neuron. NMRT not only encouraged neurite outgrowth but also enhanced neuronal maturation of individual neurons. Of note, we found that NMRT treated DRG cultures contained a high number of SCs. As SCs can produce a variety of neurotrophic and neuritogenic factors (Jessen and Mirsky, 2016; Weiss et al., 2021), we speculated that the beneficial effect of NMRT on neurons could have been indirectly mediated by the SCs or their secreted factors present in the DRG cultures. By comparing the impact of conditioned medium derived from NMRT treated and untreated SC cultures on freshly dissociated DRGs, we showed that the supernatant of treated SCs was sufficient to increase the attachment and neurite network formation of DRG neurons as early as 24 h after seeding. This finding indicates that NMRT induces the secretion of neurotrophic and neuritogenic factors in SCs, responsible for the enhanced DRG neuron survival and neurite outgrowth found in the NMRT treated DRG cultures. The responsiveness of SCs to NMRT further underlines their highly reactive cellular state (Jessen and Mirsky, 2016), but it remains to be elucidated how the NMRT input is converted into a cellular signal. Recently, an involvement of the cellular redox system was suggested to play a role (Oliva et al., 2019). Further research is necessary to validate if this finding was cell specific or could represent a general mode of action also applicable for SCs. Whether NMRT is able to directly affect the cellular behavior of neurons needs to be clarified in future studies.

Based on the increased SC number and enhanced neuronal maturation status observed in NMRT treated DRG cultures, the impact of NMRT on axon remyelination is worthwhile investigating in the future. We assume that the NMRT induced SC stimulation starts a cascade of reciprocal signaling events between SCs and the regrowing axons necessary for myelination. *In vitro* myelination experiments on DRG neuron and SC co-cultures should reveal if NMRT treatment indeed supports SC redifferentiation into a myelinating phenotype or rather retains them in a repair state. This information is important with regard to the establishment of appropriate NMRT treatment regimen to further study its effect on peripheral nerve regeneration in animal models.

Taken together, our study shows for the first time that NMRT significantly enhances the regeneration of DRG neurons *in vitro*. This effect is to a significant extent mediated by the stimulation of SCs to support DRG neuron survival and neurite outgrowth. Thus, NMRT might represent a non-invasive treatment option that could be used to support standard therapies for peripheral nerve regeneration.

## Data Availability Statement

The raw data supporting the conclusions of this article will be made available by the authors, without undue reservation.

## Ethics Statement

Ethical review and approval was not required for the animal study because according to the Austria’s Animal Testing Law (TVG 2012, §2, 1.c) and Article 3 of the Directive 2010/63/EU of the European Parliament and of the Council on the Protection of Animals Used for Scientific Purposes (European Parliament, 2010), the sole tissue harvest from sacrificed animals to obtain cell cultures does not require an ethical approval. The sacrifice of animals was conducted in compliance with Austria’s Animal Testing Law (TVG 2012, §2, 1.c) and Article 3 of the Directive 2010/63/EU of the European Parliament and of the Council on the Protection of Animals Used for Scientific Purposes (European Parliament, 2010).

## Author Contributions

AM, BS-F, and TW: conceptualization. AM, FM, SW, LM, and LW: methodology and investigation. LS, PS, and FM: material. AM and AN: software. AM and TW: visualization and writing and draft preparation. AM, AN, and TW: formal analysis. AM: project administration. TW and CR: supervision. CR, BS-F, and LW: funding. All authors: review and editing.

## Conflict of Interest

The authors declare that the research was conducted in the absence of any commercial or financial relationships that could be construed as a potential conflict of interest.

## Publisher’s Note

All claims expressed in this article are solely those of the authors and do not necessarily represent those of their affiliated organizations, or those of the publisher, the editors and the reviewers. Any product that may be evaluated in this article, or claim that may be made by its manufacturer, is not guaranteed or endorsed by the publisher.

## Footnotes

1
http://imagej.nih.gov/ij/

## Abbreviations

SCs: Schwann cells
PNS: peripheral nervous system
DRG: dorsal root ganglion
NMRT: nuclear magnetic resonance therapy
PNI: peripheral nerve injury
PBS: Dulbecco’s phosphate buffered saline
MEM ∝: Minimum Essential Medium alpha
NaP: sodium pyruvate solution
P/S: penicillin-streptomycin
MFI: mean fluorescence intensity
RT-PCR: reverse transcription polymerase chain reaction
SD: standard deviation
TBB3: tubulin β -3
NFH: neurofilament heavy polypeptide
NGF: nerve growth factor
FGF-basic: fibroblast growth factor basic
PDGF-AA: platelet-derived growth factor.
