# Supplementary material for: Nuclear Magnetic Resonance Treatment Accelerates the Regeneration of Dorsal Root Ganglion Neurons in vitro
Source: Front Cell Neurosci. 2022 Mar 28;16:859545. doi: 10.3389/fncel.2022.859545 (PMC8995532; doi:10.3389/fncel.2022.859545)
Supplement: Supplementary file 1 [file Data_Sheet_1.docx]

# Supplementary Figures


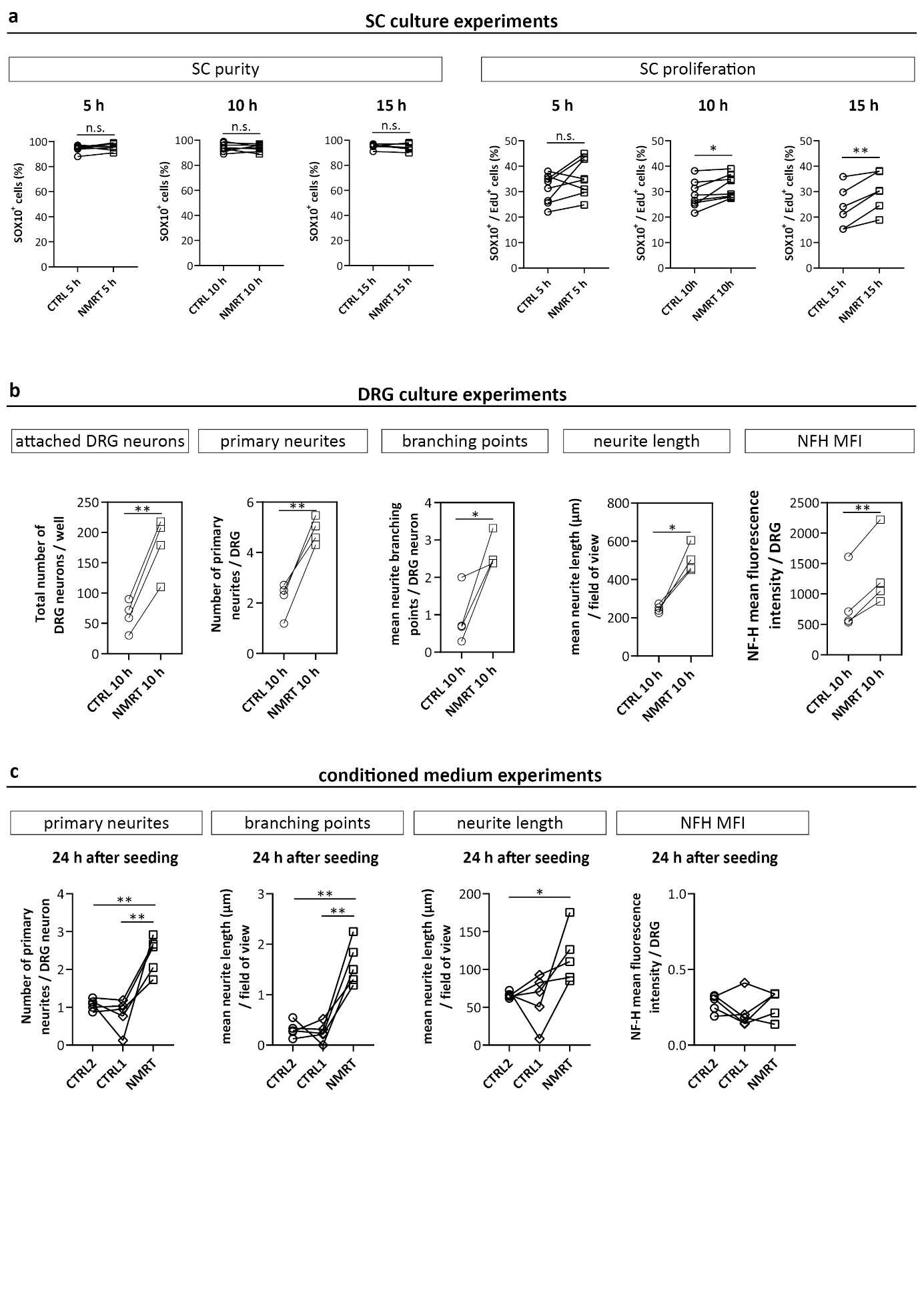


Supplementary Figure 1. Donor dependent visualization of measured parameter values for statistical analysis. (a) SC culture experiments: SC culture purity was calculated in percent of SOX10^+^/DAPI^+^ cells (SCs) from DAPI^+^ cells (all cells) and showed no difference between the NMRT and CTRL condition; data are depicted as single values for each donor ± SD (n=6), values that correspond to the same donor are connected by a line. Proliferating SCs were calculated in percent of SOX10^+^/EdU^+^ positive cells (proliferating SCs) from SOX10^+^ cells (SCs) showing an increase of EdU^+^ SCs in NMRT treated cultures after the 10 h and 15 h treatment cycle compared to respective controls; data are depicted as single values for each donor ± SD, 5 h (n=8), 10 h (n=8), 15 h (n=6), values that correspond to the same donor are connected by a line, two-way ANOVA, * p < 0.05, ** p < 0.01. (b) DRG culture experiments: Quantification of the mean number of primary neurites per DRG neuron, the mean number of neurite branching points per DRG neuron, the mean length of neurites per image (field of view), and the NFH mean fluorescence intensity (MFI) per DRG neuron; data are depicted as single values for each donor ± SD (n=4), values that correspond to the same donor are connected by a line, two-way ANOVA, ** p < 0.01, * p < 0.05. (c) Conditioned medium experiments: Quantification of the mean number of primary neurites per DRG neuron, the mean number of neurite branching points per DRG neuron, the mean length of neurites per image (field of view), and NFH mean fluorescence intensity (MFI) per DRG neuron; data are depicted as single values for each donor ± SD (n=5), values that correspond to the same donor are connected by a line, two-way ANOVA, ** p < 0.01, * p < 0.05. Conditioned medium obtained from NMRT treated SC cultures (NMRT), conditioned medium obtained from untreated SC cultures (CTRL1), and normal SC medium (CTRL2).


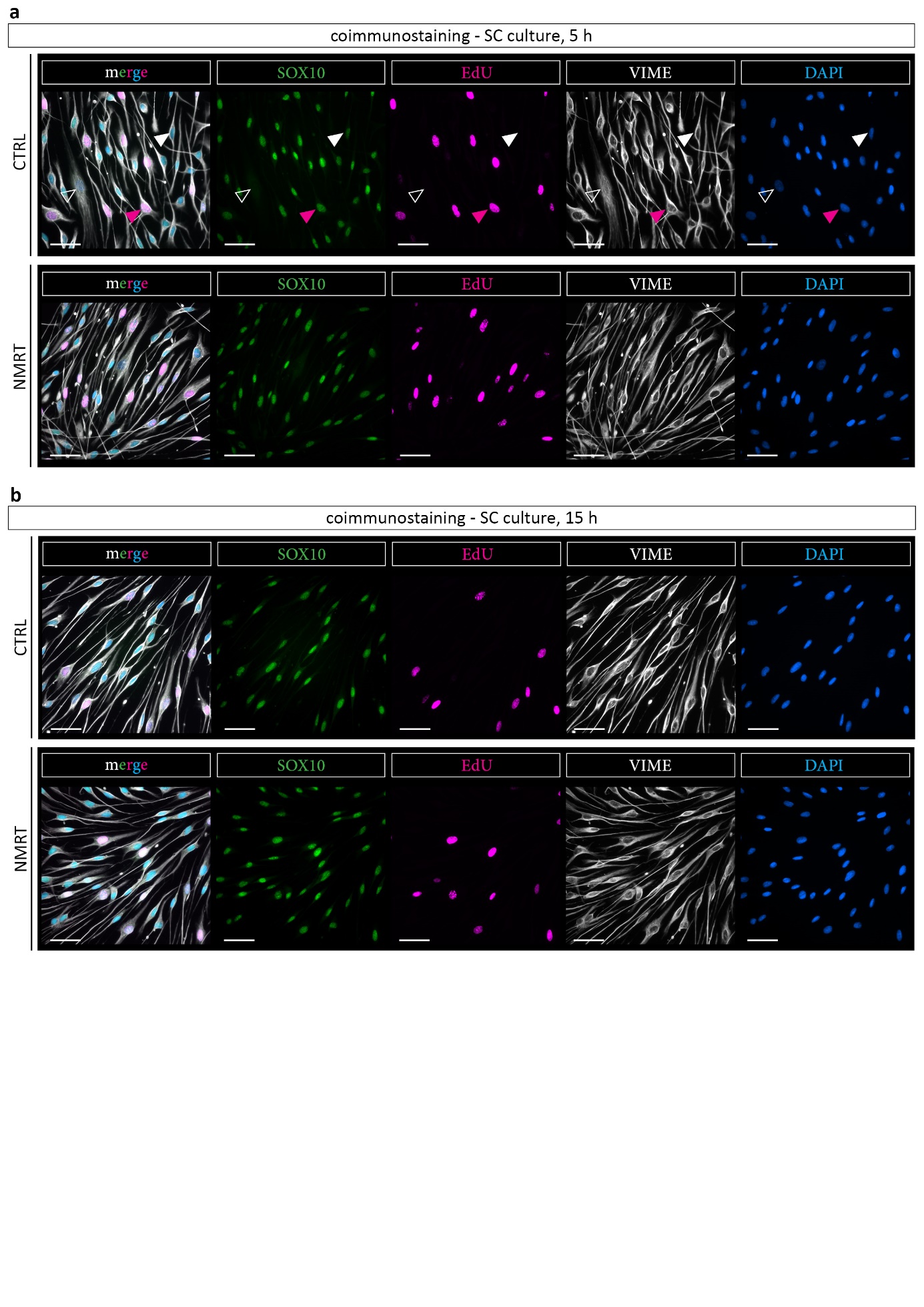


Supplementary Figure 2. Comparison of SC behavior in response to NMRT treatment. Representative fluorescence images of control and NMRT treated SCs after the 5 h (a) and 15 h (b) treatment cycles stained for SC marker SOX10 (green), proliferation marker EdU (magenta), intermediate filament vimentin (VIME, white) and DAPI (blue), filled white arrowheads indicate a SOX10^+^/EdU^-^/VIME^+^/DAPI^+^ SC, lined white arrowheads indicate a SOX10^-^/ EdU^-^/VIME^+^/DAPI^+^ fibroblast, filled magenta arrowheads indicate a SOX10^+^/EdU^+^/VIME^+^/DAPI^+^ SC. Scale bars represent 50 µm.

**Supplementary Tables**

**Supplementary Table 1: Primary and secondary antibodies**

| **Primary Antibodies** | | | | |
| --- | --- | --- | --- | --- |
| Antigen | species | Dilution | Company | Comment |
| S100 | rabbit | 1:200 | DAKO, #Z0311 | Permeabilization,  o.n. incubation at 4°C |
| Vimentin (VIME) | chicken | 1:300 | ThermoFisher, PA1-10003 | Permeabilization,  o.n. incubation at 4°C |
| SOX10 | mouse | 1:50 | Santa Cruz, sc-53116 | Permeabilization,  o.n. incubation at 4°C |
| ß3Tubulin (TBB3) | mouse | 1:100 | Santa Cruz, B2217 | Permeabilization,  o.n. incubation at 4°C |
| NFH | chicken | 1:400 | Invitrogen, PA1-10002 | Permeabilization, o.n. incubation at 4°C |
| **Secondary Antibodies** | | | | |
| Fluorophore | Target species | Dilution | Company | Comment |
| AF488 | mouse | 1:300 | Invitrogen, A11029 | 1 h incubation, RT |
| AF594 | rabbit | 1:300 | Invitrogen, A11012 | 1 h incubation, RT |
| DL650 | chicken | 1:300 | Invitrogen, SA5-10073 | 1 h incubation, RT |

**Supplementary Table 2: Primer sequences used for RT-PCR**

|  | **Oligonucleotide sequences** | |
| --- | --- | --- |
| Target gene | forward Primer | reverse Primer |
| *Gapdh* | AGTGCCAGCCTCGTCTCATA | GATGGTGATGGGTTTCCCGT |
| *Actb* | GCAGGAGTACGATGAGTCCG | ACGCAGCTCAGTAACAGTCC |
| *Ngfr* | GATTCTAGGGATGTCCTCTG | CATCGGAGAATGTAACACTG |
| *Jun* | AAACAGAAAGTCATGAACCAC | CAACCAGTCAAGTTCTCAAG |
| *ErbB3* | AATCTGGACTTCCTCATCAC | TTTAGGTAACCTGTGATCTCC |
